# The effects of chatbot characteristics and customer experience on satisfaction and continuance intention toward banking chatbots: Data from Vietnam

**DOI:** 10.1016/j.dib.2023.110025

**Published:** 2024-01-03

**Authors:** Xuan Cu Le, Tran Hung Nguyen

**Affiliations:** Department of Economic Information System and Electronic Commerce, Thuongmai University, Hanoi, 100000, Vietnam

**Keywords:** Artificial intelligence-powered chatbots, Behavioural outcomes, Extrinsic value, Intrinsic value, Understandability, Intrusiveness

## Abstract

The article depicts the dataset of a survey on the effects of chatbot characteristics on customer experience (including intrinsic and extrinsic values) and behavioural outcomes (including satisfaction and continuance intention) toward chatbots in the context of banking within Vietnam. The data were accumulated using a web-based questionnaire with a valid sample of 336 participants who have used banks’ chatbots in Vietnam from July 2023 to September 2023. Participants were encouraged to share the survey link with different chatbot users via social media to seek potential respondents. Harman single factor was utilized to lessen the issue of common method bias. The formal data were evaluated by using SPSS 21.0 and AMOS 21.0. In addition to respondents’ demographic profile, the results of explanatory factor analysis and confirmation factor analysis were presented in this work, which would alluded as a good reference for future studies.

Specifications Table

**Table:** 

Subject	Management of Technology and Innovation.
Specific subject area	Digital technology in marketing applications, Electronic commerce, Consumer behaviour in digital transformation, Consumer experience and digital strategies, Finance and banking*.*
Data format	Raw, Analyzed
Type of data	Table
Data collection	Survey Questionnaire (included in Supplementary Materials).
Data source location	Respondents are voluntary customers who have used chatbot-based banking services in Vietnam banks.
Data accessibility	Repository name: Open Science Framework [1] Data identification number: https://doi.org/10.17605/OSF.IO/6QPBH Direct URL to data: https://doi.org/10.17605/OSF.IO/6QPBH

## 1 Value of the Data

• The dataset disseminates the knowledge about the vital role of artificial intelligence-powered chatbots in boosting customers’ new experience (e.g., conveying useful information, solving deficiencies, streamlining time-consuming tasks, providing personalized banking services, supporting customer care, and handling financial transactions) without much human resources and in fostering behavioural outcomes (e.g., satisfaction and prolonged use) toward banking chatbots.
• The data are meaningful for academia and practitioners (i.e., banks and financial institutes) to understand how chatbots make reciprocity with customers, enhance bank-customer relationship, and promote sustainable development of banking in Vietnam.
• The data can be reused for researchers who show their increasing interests in the topic of digital technology like artificial intelligence-powered chatbots and its motivation for customer experience and behavioural outcomes in Vietnam and in other emerging markets.
• The dataset is a valuable reference source for research on smart technology in marketing applications as well as consumer perceptions and behaviours toward chatbots.

## 2 Background

Digital transformation has diffused widely digital technologies in banking, which results in the change in business models in reinforcing customer experience and dealing with information and services [2]. It is necessary for banks to increase business sustainability due to digital technologies, such as artificial intelligence-powered chatbots. Chatbots become a powerful instrument for banks to strengthen the interaction with customers, leading to enhancing service quality, cementing bank-customer reciprocity, and promoting customer experience [3]. Past studies documented that customer experience is driven by chatbot characteristics and this experience fosters post-adoption behaviours, including satisfaction and continuance intention [4]. Furthermore, following the information system success model [5], content quality, system quality and service quality are core determinants of satisfaction and continuance intention. Hence, content quality, system quality (interaction, competence, and automation), and service quality (personalization, understandability, intimacy, and intrusiveness) could facilitate customer experience, which, in turn, affects behavioural outcomes (satisfaction and continuance intention) toward banking chatbots.

## 3 Data Description

The survey was developed to gather empirical data about twelve constructs, namely content quality, interaction, competence, personalization, automation, understandability, intimacy, intrusiveness, intrinsic value, extrinsic value, satisfaction, and continuance intention. Items of these constructs were selected from extant studies and slightly modified to fit the current context. First, content quality (four items, Cronbach's alpha = 0.940) was assessed via four criteria: Content provided by chatbots is accurate (COQ1), Content provided by chatbots is sufficiently timely (COQ2), Content provided by chatbots is relevant to my decision-making (COQ3), Chatbots provide content pertaining to my concerns (COQ4). Second, interaction (three items, Cronbach's alpha = 0.896) was assessed via three criteria: I am in control of my personal needs through chatbots (ACT1), I perceive chatbots to be sensitive to my personal needs (ACT2), Chatbots provide opportunities to give my responses (ACT3). Third, competence (three items, Cronbach's alpha = 0.904) was assessed via three criteria: Chatbots are competent (COM1), Chatbots are intelligent (COM2), Chatbots are skillful (COM3). Fourth, personalization (three items, Cronbach's alpha = 0.957) was assessed via three criteria: Chatbots understand my needs (PER1), Chatbots know what I want (PER2), The advice appears to tailored for me personally (PER3). Fifth, automation (four items, Cronbach's alpha = 0.887) was assessed via four criteria: It is convenient that chatbots help users proactively without human intervention (AUT1), It is convenient that chatbots provide auto-adjusted control (AUT2), Chatbots autonomously provide me the choice of what to do (AUT3), Chatbots independently provide me recommendations for action plans for assigned matters (AUT4). Sixth, understandability (three items, Cronbach's alpha = 0.907) was assessed via three criteria: I feel that what I am saying to chatbots is well understood by the system (UND1), I feel that the words in my questions are well understood by chatbots (UND2), I feel that chatbots understand my intentions when I ask questions to it (UND3). Seventh, intimacy (three items, Cronbach's alpha = 0.920) was assessed via three criteria: I develop a sense of familiarity with chatbots (INT1), Chatbots use supportive statements to build favor with me (INT2), I feel emotionally close to chatbots (INT3). Eighth, intrusiveness (three items, Cronbach's alpha = 0.925) was assessed via three criteria: While receiving responses from chatbots, I feel I am under surveillance (TRU1), While receiving responses from chatbots, I feel I am being monitored (TRU2), While receiving responses from chatbots, I feel they are listening to everything around me (TRU3). Ninth, intrinsic value (three items, Cronbach's alpha = 0.912) was assessed via three criteria: I like chatbots when they help me customize my financial experience to my own liking (IVA1), I enjoy getting the benefits from using chatbots with little effort (IVA2), Chatbots are fun to converse with (IVA3). Tenth, extrinsic value (three items, Cronbach's alpha = 0.916) was assessed via three criteria: Chatbots make me feel that they are talking to me personally as a customer (EVA1), Chatbots help resolve my needs without other problems (EV2), Chatbots make me feel valued as a customer (EVA3). Eleventh, satisfaction (four items, Cronbach's alpha = 0.884) was assessed via four criteria: I am pleased with using chatbots (SAT1), I like to use chatbots from the bank websites (SAT2), I think that using chatbots on the bank website is a good idea (SAT3), Overall, I am satisfied with the experience of using chatbots (SAT4). Lastly, continuance intention (three items, Cronbach's alpha = 0.913) was assessed via three criteria: My intention is to continue using chatbots over other alternative means of communication or searching tools on the bank websites (COI1), All things considered, I expect to continue using chatbots often in the future (COI2), I can see myself increasing the use of chatbots if possible (COI3).

Convenience sampling was used as the survey tool. We applied this method due to the absence of a reliable list of participants and its advantages including cost-time saving, quickly data collection, ease of use, and widespread adoption in behaviour science studies [4]. Given that the survey targeted respondents who have used chatbots in banks in Vietnam where there were the rising rates of mobile payment [4]. We provided an introduction of the research purpose and ensured the anonymity and privacy of participants’ responses. To enhance participants’ understanding of chatbots, we dispersed several demonstrations (including pictures and videos) before filling in the survey. A screening question about “Have you used chatbots in banking before” in the survey was added to filter the desired sample. Respondents had options for their participation or withdrawal during the surveyed time.

The questionnaire consisted of two main sections: respondent demographic-related information and measurement scales. The first section provided the detailed profile of respondents, comprising gender (2 categories: male and female), age (four categories: below 18, 18–30, 31–40, and over 40), education (3 categories: high school, undergraduate, graduate and above), occupation (4 categories: student, working, unemployed, and other), and frequency of using banks’ chatbot services in past three months (4 categories: once, twice, thrice, and more than thrice).

A total of 361 responses were returned from July to September 2023, resulting in a 92% response rate. After carefully scrutinizing all the responses for each question, 25 responses were excluded owing to missing data and duplicated answers. 336 valid answers were yielded and were subjected to further analysis. More than a half of the respondents were male (57.14%) as compared with few female (42.86%). The majority of the respondents were in the age group of 31–40 (37.80%) and 18–30 years old (33.33%). The over-40-year-old group accounted for 22%, and the under-18-year-old segment was the rest (6.8%). In terms of education, over 53.27% acquired an undergraduate degree, following by 37.80% for graduate and above degree and 8.93% for high school degree. For occupation, 53.57% have had a job, following by student (38.10%), unemployment (6.25%), and other (2.08%). Of the respondents, about 58.63% have utilized chatbots for twice in past 3 months, compared to 22.92% for once, 13.99% for thrice, and 4.46% for more than thrice. Respondents’ demographics are shown in Table 1.

**Table 1:** Respondents’ demographics (*N* = 336).

Coding in survey	Demographic		Frequency	Percentage (%)
Q1	*Gender*	Male	192	57.1
		Female	144	42.9
Q2	*Age*	< 18	23	6.8
		18–30	112	33.3
		31–40	127	37.8
		> 40	74	22
Q3	*Education*	High school	30	8.9
		Undergraduate	179	53.3
		Graduate and above	127	37.8
Q4	*Occupation*	Student	128	38.1
		Working	180	53.6
		Unemployed	21	6.3
		Other	7	2.1
Q5	*Frequency of using banks’ chatbot services in past three months*	Once	77	22.9
		Twice	197	58.6
		Thrice	47	14
		More than thrice	15	4.5

Response bias affects item validity, reliability, and the covariation between constructs, which leads to the limitation of the generalizability of outcomes. Some reasons behind response bias were considered such as respondents’ capability, insufficient experience thinking regarding the topic, sophisticated questions, and item ambiguity. In this study, convenience sampling was recruited as the survey instrument. We applied this method because the absence of a reliable list of participants and its advantages including cost-time saving, quickly data collection, ease of use, and widespread adoption in behaviour science studies [4]. However, there are some disadvantages of this sampling method, including bias in sampling, lack of variety, and possibility of researcher bias. In the study, the sample population seemed biased toward male respondents as the large proportion of the respondents were male (57.14%) as compared with few female (42.86%). Each person who agreed to participate was asked a screening question to determine whether they were actual users of banking chatbots. Most of male respondents answered “Yes” due to the familiarity with new digital technologies (e.g., chatbots) in banking compared to female respondents. Therefore, the bias in our population was identified as a limitation and should be solved in further studies.

Furthermore, some precautionary measures were conducted to address these issues. First, we ensured the privacy and confidentiality of participants’ responses. Second, the clarification of all the items of the constructs were provided by performing pre-test. Nine experts were invited to provide some suggestions about format, length, and content of the questionnaire. Third, to curb the common method bias (CMB), Harman single factor (HSF) was conducted to CMB on single respondents in the survey using SPSS 21.0 software. The result illustrated that HSF value with principal axis factor is 36.227% (< 50%) of the total variance [6]. Thus, there is no issue on CMB in the dataset.

In this study, SPSS 21.0 software was used to perform exploratory factor analysis (EFA) applying principal axis factoring with promax rotation to examine construct validity of measures. The results revealed that the Kaiser-Meyer-Olkin (KMO) value is 0.914 (> 0.50) and Barlett's test is significant at 0.000. Through the role of the Eigen value, factors are extracted and have factor loadings of each item of more than 0.05 (see Table 2).

**Table 2:** Descriptive and exploratory factor analysis results.

Variable	Mean	SD	Factor loading in the Exploratory Factor Analysis											
			COQ	ACT	COM	AUT	PER	UND	INT	TRU	EVA	IVA	SAT	COI
*Content quality (COQ)*														
COQ1	3.39	1.076	0.847											
COQ2	3.45	1.000	0.905											
COQ3	3.47	1.084	0.890											
COQ4	3.39	1.036	0.915											
*Interaction (ACT)*														
ACT1	3.31	1.144		0.740										
ACT2	3.29	1.183		0.843										
ACT3	3.29	1.194		0.921										
*Competence (COM)*														
COM1	3.43	1.066			0.772									
COM2	3.39	1.059			0.882									
COM3	3.45	1.033			0.924									
*Automation (AUT)*														
AUT1	3.31	0.931				0.869								
AUT2	3.36	0.979				0.803								
AUT3	3.37	1.026				0.839								
AUT4	3.40	1.020				0.735								
*Personalization (PER)*														
PER1	3.38	1.135					0.943							
PER2	3.36	1.086					0.914							
PER3	3.34	1.098					0.926							
*Understandability (UND)*														
UND1	3.36	1.042						0.836						
UND2	3.47	1.013						0.807						
UND3	3.45	1.041						0.898						
*Intimacy (INT)*														
INT1	3.33	1.037							0.908					
INT2	3.41	0.994							0.877					
INT3	3.39	1.037							0.878					
*Intrusiveness (TRU)*														
TRU1	3.14	1.136								0.867				
TRU2	3.20	1.095								0.928				
TRU3	3.15	1.084								0.894				
*Intrinsic value (IVA)*														
IVA1	3.39	1.068									0.886			
IVA2	3.40	1.060									0.759			
IVA3	3.46	1.039									0.937			
*Extrinsic value (EVA)*														
EVA1	3.51	1.042										0.839		
EVA2	3.43	1.034										0.879		
EVA3	3.43	1.066										0.890		
*Satisfaction (SAT)*														
SAT1	3.50	0.860											0.734	
SAT2	3.48	0.864											0.858	
SAT3	3.52	0.917											0.841	
SAT4	3.51	0.807											0.775	
*Continuance intention (COI)*														
COI1	3.79	1.061												0.823
COI2	4.01	1.106												0.898
COI3	4.01	1.064												0.926

The results of descriptive statistics showed that the mean of variables is between 3.14 and 4.01, and the standard deviation is between 0.807 and 1.194. Specifically, items of continuance intention reached the largest mean among variables, ranging from 3.77 to 4.01, whereas items of intrusiveness had the lower mean than of the remaining items of other variables, ranging from 3.14 to 3.20. Additionally, items of interaction had the highest standard deviation, ranging from 1.144 to 1.197, whilst items of satisfaction had the lowest standard deviation, ranging from 0.807 to 0.917 (see Table 2).

Furthermore, the confirmation factor analysis demonstrated a good model fit for the model as demonstrated that χ*^2^*/df = 0.149 (< 3.0), TLI = 0.967 (≥ 0.90), CFI = 0.971(≥ 0.90), NFI = 0.917 (≥ 0.90), IFI = 0.971 (≥ 0.90); and RMSEA = 0.038 (< 0.08) [7]. Additionally, the study assessed the reliability of the measures through Cronbach's alpha (CA). As seen in Table 3, CA values of all the variables met the threshold of more than 0.70 [7].

**Table 3:** Confirmation factor analysis results.

Coding in survey	Constructs & items		Std-loading
*Content quality – Adapted from Jung* et al. *[**9**] (Cronbach's alpha = 0.940; AVE = 0.797; CR = 0.940)*			
Q6	COQ1	Content provided by chatbots is accurate	0.890
Q7	COQ2	Content provided by chatbots is sufficiently timely	0.885
Q8	COQ3	Content provided by chatbots is relevant to my decision-making	0.903
Q9	COQ4	Chatbots provide content pertaining to my concerns	0.894
*Interaction – Adapted from Cho* et al. *[**10**] (Cronbach's alpha = 0.896; AVE = 0.745; CR = 0.897)*			
Q10	ACT1	I am in control of my personal needs through chatbots	0.839
Q11	ACT2	I perceive chatbots to be sensitive to my personal needs	0.846
Q12	ACT3	Chatbots provide opportunities to give my responses	0.903
*Competence – Adapted from Cuddy* et al. *[**11**] (Cronbach's alpha = 0.904; AVE = 0.758; CR = 904)*			
Q13	COM1	Chatbots are competent	0.868
Q14	COM2	Chatbots are intelligent	0.872
Q15	COM3	Chatbots are skillful	0.872
*Automation – Adapted from Luor* et al. *[**12**] (Cronbach's alpha = 0.887; AVE = 0.668; CR = 0.889)*			
Q16	AUT1	It is convenient that chatbots help users proactively without human intervention	0.860
Q17	AUT2	It is convenient that chatbots provide auto-adjusted control	0.819
Q18	AUT3	Chatbots autonomously provide me the choice of what to do	0.823
Q19	AUT4	Chatbots independently provide me recommendations for action plans for assigned matters	0.764
*Personalization – Adapted from Zhang and Curley [**13**] (Cronbach's alpha = 0.957; AVE = 0.881; CR = 0.957)*			
Q20	PER1	Chatbots understand my needs	0.930
Q21	PER2	Chatbots know what I want	0.938
Q22	PER3	The advice appears to tailored for me personally	0.948
*Understandability – Adapted from Li* et al. *[**14**] (Cronbach's alpha = 0.907; AVE = 0.765; CR = 0.907)*			
Q23	UND1	I feel that what I am saying to chatbots is well understood by the system	0.904
Q24	UND2	I feel that the words in my questions are well understood by chatbots	0.835
Q25	UND3	I feel that chatbots understand my intentions when I ask questions to it	0.883
*Intimacy – Adapted from Berscheid* et al. *[**15**] (Cronbach's alpha = 0.920; AVE = 0.793; CR = 0.920)*			
Q26	INT1	I develop a sense of familiarity with chatbots	0.905
Q27	INT2	Chatbots use supportive statements to build favor with me	0.870
Q28	INT3	I feel emotionally close to chatbots	0.896
*Intrusiveness – Adapted from Lau* et al. *[**16**] (Cronbach's alpha = 0.925; AVE = 0.807; CR = 0.926)*			
Q29	TRU1	While receiving responses from chatbots, I feel I am under surveillance	0.887
Q30	TRU2	While receiving responses from chatbots, I feel I am being monitored	0.910
Q31	TRU3	While receiving responses from chatbots, I feel they are listening to everything around me	0.897
*Intrinsic value – Adapted from Roy* et al. *[**17**] (Cronbach's alpha = 0.912; AVE = 0.780; CR = 0.914)*			
Q32	IVA1	I like chatbots when they help me customize my financial experience to my own liking	0.890
Q33	IVA2	I enjoy getting the benefits from using chatbots with little effort	0.835
Q34	IVA3	Chatbots are fun to converse with	0.923
*Extrinsic value – Adapted from Rose* et al. *[**18**] (Cronbach's alpha = 0.916; AVE = 0.785; CR = 0.916)*			
Q35	EVA1	Chatbots make me feel that they are talking to me personally as a customer	0.873
Q36	EVA2	Chatbots help resolve my needs without other problems	0.902
Q37	EVA3	Chatbots make me feel valued as a customer	0.883
*Satisfaction – Adapted from Fang* et al. *[**19**] (Cronbach's alpha = 0.884; AVE = 0.658; CR = 0.885)*			
Q38	SAT1	I am pleased with using chatbots	0.781
Q39	SAT2	I like to use chatbots from the bank websites	0.802
Q40	SAT3	I think that using chatbots on the bank website is a good idea	0.854
Q41	SAT4	Overall, I am satisfied with the experience of using chatbots	0.805
*Continuance intention – Adapted from Bhattacherjee [**20**] (Cronbach's alpha = 0.913; AVE = 0.782; CR = 0.915)*			
Q42	COI1	My intention is to continue using chatbots over other alternative means of communication or searching tools on the bank websites	0.835
Q43	COI2	All things considered, I expect to continue using chatbots often in the future	0.905
Q44	COI3	I can see myself increasing the use of chatbots if possible	0.911

For convergent validity, we employed two criteria: composite reliability (CR) and average variance extracted (AVE). The results indicated that AVE values exceed the 0.50 cut-off (between 0.658 and 0.881) and CR value exceed the 0.07 threshold (between 0.885 and 0.957) (see Table 3).

For discriminant validity, we conducted the procedure recommended by Fornell and Larker [8]. As seen in Table 4, the square root values of AVE (bold diagonal) of the constructs (from 0.811 to 0.939) were greater than all the coefficient correlations between the corresponding constructs.

**Table 4:** Discriminant validity.

	COQ	ACT	COM	AUT	PER	UND	INT	TRU	EVA	IVA	SAT	COI
COQ	**0.893**											
ACT	0.396	**0.863**										
COM	0.484	0.557	**0.871**									
AUT	0.255	0.403	0.355	**0.817**								
PER	0.457	0.599	0.537	0.409	**0.939**							
UND	0.460	0.632	0.591	0.378	0.660	**0.875**						
INT	0.280	0.409	0.367	0.346	0.322	0.348	**0.891**					
TRU	0.246	0.244	0.280	0.231	0.272	0.259	0.164	**0.898**				
EVA	0.439	0.543	0.523	0.414	0.322	0.565	0.322	0.249	**0.886**			
IVA	0.444	0.564	0.534	0.386	0.549	0.595	0.414	0.133	0.473	**0.883**		
SAT	0.554	0.361	0.416	0.255	0.459	0.461	0.226	0.144	0.381	0.376	**0.811**	
COI	0.510	0.571	0.654	0.368	0.597	0.623	0.340	0.253	0.591	0.631	0.613	**0.884**

*Notes:* Diagonal values (in bold) – demonstrate the square root of AVE of the construct.

## 4 Experimental Design, Materials and Methods

All constructs in the survey were measured using scales adapted from current literature (see Table 3). These scales were slightly amended based on the given setting of banking chatbots. Content quality (four items) was adapted from Jung et al. [9] and interaction (three items) was adapted from Cho et al. [10]. Three items measuring competence were adapted from Cuddy et al. [11]. Four items from Luor et al. [12] were used to measure automation. Personalization (three items) was adapted from Zhang and Curley [13], whereas understandability (three items) and intimacy (three items) were adapted from Li et al. [14] and Berscheid et al. [15] respectively. Three items measuring intrusiveness were adapted from Lau et al. [16]. Intrinsic value (three items) was adapted from Roy et al. [17] and extrinsic value (three items) was adapted from Rose et al. [18]. Furthermore, satisfaction was measured by four items which were adapted from Fang et al. [19], while continuance intention was measured by three items which were adapted from Bhattacherjee [20]. All items were anchored on an appropriately labeled five-point Likert scale 1 – strongly disagree, 2 – disagree, 3 – neutral, 4 – agree, 5 – strongly agree.

Based on national language, English questionnaire was translated into Vietnamese through the assistance of a marketing expert. The Vietnamese version then was translated back into English by another expert and carefully checked to lessen the distinction compared to the original English version. The questionnaire was designed using Google Forms. Facebook was selected to spread the questionnaire to participants because it is one of the influential social media platforms on Vietnamese people’ daily basis [4]. 361 responses were gathered from July to September 2023. After carefully scrutinizing all the responses for each question, 25 responses were excluded owing to missing data and duplicated answers. A total of 336 valid responses remained valid for final analysis.

Data were gathered in a two-stage process. First, the questionnaire was tailored due to extant validated studies. We discussed the questionnaire with nine experts specialized in banking and information system (one full professor, five associate professors, and three Ph.Ds) to ensure validity of all the constructs. Consequently, some main amendments of the items were suggested including content quality, automation, intimacy, and intrusiveness. Next, a pilot study was conducted with a small sample (36 respondents). The results confirmed that coefficient alpha values of variables exceeded 0.7 [7], thereby construct validity is satisfactory. Data in the pilot survey were not shown in the final survey. Consequently, 39 items of 12 constructs were retained.

## Limitations

Not applicable.

## Ethics Statement

Before participating in this study, all respondents were fully briefed on its nature and the research purpose. All subjects gave their informed consent for inclusion before filling out the questionnaire. Specifically, with some minor participants aged under 18, information consent was obtained from these respondents and their parents. Data were offered anonymised. Moreover, this work met Elsevier ethical publishing requirements, including (https://www.sciencedirect.com/journal/data-in-brief/publish/guide-for-authors) and (https://www.elsevier.com/about/policies-and-standards/publishing-ethics#Authors).

## CRediT authorship contribution statement

**Xuan Cu Le:** Conceptualization, Writing – review & editing, Funding acquisition, Software, Formal analysis, Data curation, Writing – original draft, Supervision. **Tran Hung Nguyen:** Conceptualization, Writing – review & editing, Funding acquisition, Methodology, Validation, Investigation, Resources, Visualization, Project administration.

## Data Availability

Banking chatbots in VN (Original data) (Open Science Framework)

## Appendix Supplementary materials

Image, application 1
