# Supplementary material for: The effects of chatbot characteristics and customer experience on satisfaction and continuance intention toward banking chatbots: Data from Vietnam
Source: Data Brief. 2024 Jan 3;52:110025. doi: 10.1016/j.dib.2023.110025 (PMC10801293; doi:10.1016/j.dib.2023.110025)
Supplement: Supplementary file 1 [file mmc1.doc]

**THE QUESTIONNAIRE**

**THE TOPIC: THE EFFECTS OF CHATBOT CHARACTERISTICS AND CUSTOMER EXPERIENCE ON SATISFACTION AND CONTINUANCE INTENTION TOWARD BANKING CHATBOTS**

Dear Sir/Madam,

Our team has being conducted an academic study entitled “*The effects of chatbot characteristics and customer experience on satisfaction and continuance intention toward banking chatbots*” in Vietnam. We would like to gain a holistic understanding of the importance of chatbot in the field of banking as well as it influences on Vietnamese customer experience and behaviors. Thus, it is necessary for us to receive your considerable support and validated responses through a survey.

**Section 1. Respondents’ profile:** (*Tick
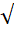
 in the best option*)

| **Q1** | Gender: | 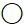 Male 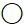 Female |
| --- | --- | --- |
| **Q2** | Age: | 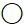 < 18 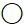 18−30 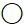 31−40 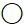 > 40 |
| **Q3** | Education: | 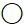 High school 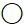 Undergraduate 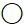 Graduate and above |
| **Q4** | Occupation: | 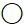 Student 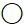 Working 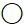 Unemployed 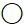 Other |
| **Q5** | *Frequency of using banks’ chatbot services in past three months*: | 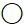 Once 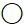 Twice 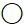 Thrice 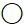 More than thrice |

**Section 2. MEASUREMENT SCALES:** (*State the extent to which you agree or disagree with each of the following statements*) (**1 – Strongly disagree, 2 – Disagree, 3 – Neutral, 4 – Agree; 5 – Strongly agree**)

|  |  |  | **1** | **2** | **3** | **4** | **5** |
| --- | --- | --- | --- | --- | --- | --- | --- |
|  | ***Content quality*** | | | | | | |
| **Q6** | COQ1 | Content provided by chatbots is accurate | 1 | 2 | 3 | 4 | 5 |
| **Q7** | COQ2 | Content provided by chatbots is sufficiently timely | 1 | 2 | 3 | 4 | 5 |
| **Q8** | COQ3 | Content provided by chatbots is relevant to my decision-making | 1 | 2 | 3 | 4 | 5 |
| **Q9** | COQ4 | Chatbots provide content pertaining to my concerns | 1 | 2 | 3 | 4 | 5 |
|  | ***Interaction*** | | | | | | |
| **Q10** | ACT1 | I am in control of my personal needs through chatbots | 1 | 2 | 3 | 4 | 5 |
| **Q11** | ACT2 | I perceive chatbots to be sensitive to my personal needs | 1 | 2 | 3 | 4 | 5 |
| **Q12** | ACT3 | Chatbots provide opportunities to give my responses | 1 | 2 | 3 | 4 | 5 |
|  | ***Competence*** | | | | | | |
| **Q13** | COM1 | Chatbots are competent | 1 | 2 | 3 | 4 | 5 |
| **Q14** | COM2 | Chatbots are intelligent | 1 | 2 | 3 | 4 | 5 |
| **Q15** | COM3 | Chatbots are skillful | 1 | 2 | 3 | 4 | 5 |
|  | ***Automation*** | | | | | | |
| **Q16** | AUT1 | It is convenient that chatbots help users proactively without human intervention | 1 | 2 | 3 | 4 | 5 |
| **Q17** | AUT2 | It is convenient that chatbots provide auto-adjusted control | 1 | 2 | 3 | 4 | 5 |
| **Q18** | AUT3 | Chatbots autonomously provide me the choice of what to do | 1 | 2 | 3 | 4 | 5 |
| **Q19** | AUT4 | Chatbots independently provide me recommendations for action plans for assigned matters | 1 | 2 | 3 | 4 | 5 |
|  | ***Personalization*** | | | | | | |
| **Q20** | PER1 | Chatbots understand my needs | 1 | 2 | 3 | 4 | 5 |
| **Q21** | PER2 | Chatbots know what I want | 1 | 2 | 3 | 4 | 5 |
| **Q22** | PER3 | The advice appears to tailored for me personally | 1 | 2 | 3 | 4 | 5 |
|  | ***Understandability*** | | | | | | |
| **Q23** | UND1 | I feel that what I am saying to chatbots is well understood by the system | 1 | 2 | 3 | 4 | 5 |
| **Q24** | UND2 | I feel that the words in my questions are well understood by chatbots | 1 | 2 | 3 | 4 | 5 |
| **Q25** | UND3 | I feel that chatbots understand my intentions when I ask questions to it | 1 | 2 | 3 | 4 | 5 |
|  | ***Intimacy*** | | | | | | |
| **Q26** | INT1 | I develop a sense of familiarity with chatbots | 1 | 2 | 3 | 4 | 5 |
| **Q27** | INT2 | Chatbots use supportive statements to build favor with me | 1 | 2 | 3 | 4 | 5 |
| **Q28** | INT3 | I feel emotionally close to chatbots | 1 | 2 | 3 | 4 | 5 |
|  | ***Intrusiveness*** | | | | | | |
| **Q29** | TRU1 | While receiving responses from chatbots, I feel I am under surveillance | 1 | 2 | 3 | 4 | 5 |
| **Q30** | TRU2 | While receiving responses from chatbots, I feel I am being monitored | 1 | 2 | 3 | 4 | 5 |
| **Q31** | TRU3 | While receiving responses from chatbots, I feel they are listening to everything around me | 1 | 2 | 3 | 4 | 5 |
|  | ***Intrinsic value*** | | | | | | |
| **Q32** | IVA1 | I like chatbots when they help me customize my financial experience to my own liking | 1 | 2 | 3 | 4 | 5 |
| **Q33** | IVA2 | I enjoy getting the benefits from using chatbots with little effort | 1 | 2 | 3 | 4 | 5 |
| **Q34** | IVA3 | Chatbots are fun to converse with | 1 | 2 | 3 | 4 | 5 |
|  | ***Extrinsic value*** | | | | | | |
| **Q35** | EVA1 | Chatbots make me feel that they are talking to me personally as a customer | 1 | 2 | 3 | 4 | 5 |
| **Q36** | EVA2 | Chatbots help resolve my needs without other problems | 1 | 2 | 3 | 4 | 5 |
| **Q37** | EVA3 | Chatbots make me feel valued as a customer | 1 | 2 | 3 | 4 | 5 |
|  | ***Satisfaction*** | | | | | | |
| **Q38** | SAT1 | I am pleased with using chatbots | 1 | 2 | 3 | 4 | 5 |
| **Q39** | SAT2 | I like to use chatbots from the bank websites | 1 | 2 | 3 | 4 | 5 |
| **Q40** | SAT3 | I think that using chatbots on the bank website is a good idea | 1 | 2 | 3 | 4 | 5 |
| **Q41** | SAT4 | Overall, I am satisfied with the experience of using chatbots | 1 | 2 | 3 | 4 | 5 |
|  | ***Continuance intention*** | | | | | | |
| **Q42** | COI1 | My intention is to continue using chatbots over other alternative means of communication or searching tools on the bank websites | 1 | 2 | 3 | 4 | 5 |
| **Q43** | COI2 | All things considered, I expect to continue using chatbots often in the future | 1 | 2 | 3 | 4 | 5 |
| **Q44** | COI3 | I can see myself increasing the use of chatbots if possible | 1 | 2 | 3 | 4 | 5 |

Thank you for your answers!
